# Supplementary material for: Agent-based model demonstrates the impact of nonlinear, complex interactions between cytokines on muscle regeneration
Source: eLife. 2024 Jun 3;13:RP91924. doi: 10.7554/eLife.91924 (PMC11147512; doi:10.7554/eLife.91924)
Supplement: Supplementary file 1. [file elife-91924-supp1.docx]

**Supplemental Table 1.** Unknown model parameters calibrated using LHS to recapitulate published literature

| Parameter | Description | Range Tested | Value |
| --- | --- | --- | --- |
| neutroRecruitmentProportion | Number of neutrophils generated at every recruitment as proportion of necrotic cells | (4, 12) | 7.8322 |
| secretionUptakeValNeu | Cytokine secreted by neutrophil per step during and after "eating necrosis" also max uptake value | (20, 80) | 37.3001 |
| necrosisThreshold | Neutrophils are recruited as long as the necrosis amount is greater than this parameter | (0.75, 0.98) | 0.9202 |
| macroRecruitmentProportion | Recruitment of macrophages is proportional to mean MCP-1 | (0.1, 0.7) | 0.4701 |
| secretionUptakeValMac | Cytokine secreted by macrophages per step, also max uptake value | (0.3, 95) | 48.2090 |
| TNFconverter | Value of TNF-α to convert monocyte to M1 | (15, 150) | 109.9025 |
| macRecruitThres | Required mean MCP-1 to signal recruitment of macrophage | (0.05, 4.5) | 1.4289 |
| macRecruitStop | Required mean TGF-β to signal stop of macrophage recruitment | (0.05, 0.4) | 0.1406 |
| IL10TransThreshold | Local IL-10 that can induce M1 to M2 transition | (15, 600) | 78.5442 |
| phagoThresholdforNeuM2 | Phagocytosis threshold for neutrophil apoptosis or M1 to M2 transition | (0, 8) | 2.0706 |
| sscActivationThreshold | Level of HGF to activate SSC | (10, 100) | 44.7091 |
| recruitmentProportionSSC | Number of SSC recruited every time step is proportional to sum of HGF + MMP-9 – TGF-β means | (0.08, 1) | 0.8213 |
| quiescentProb | Probability of SSC apoptosis vs remaining quiescent | (1, 4) | 3.3617 |
| secretionUptakeValSSC | Cytokine secreted by SSC per step when activated also max uptake value | (0.5, 200) | 44.4650 |
| sscTGFApoptosisThreshold | Amount of TGF-β to induce apoptosis | (50, 190) | 79.1563 |
| sscApoptosisProb | Probability of SSC apoptosis with apoptosis signal | (0.2, 0.99) | 0.7651 |
| VEGFblockApop | Amount of VEGF-A required to block SSC apoptosis | (60, 190) | 80.6055 |
| SSCdivisionThreshold | TNF-α + VEGF-A – TGF-β has to be greater than this for SSC to divide | (50, 400) | 126.6268 |
| SSCdiffThreshold | IL-10 – HGF – TNF-α – TGF-β has to be greater than this for SSC diff | (5, 145) | 57.9870 |
| sscDivideProb | Probability of SSC to divide if signal is not present | (0.01, 0.6) | 0.0972 |
| sscDiffProb | Probability for SSC to differentiate without signal | (0.06, 1) | 0.6136 |
| fuseProb | Probability of SSC fusing even if the collagen amount is still low | (0.06, 1) | 0.3 |
| quiescentThreshold | SSC will return to quiescence if HGF is lower than this at their location | (0.2, 100) | 36.3109 |
| necrosisSecretionVal | Amount of HGF and TGF-β secreted by necrotic cells per step | (0.0005, 35) | 21.9514 |
| VEGFsecretionValFib | VEGF-A secreted by fibers per step | (0.02, 0.1) | 0.0498 |
| VEGF_MMPthreshold | Level of VEGF-A to trigger sprouting/elongation from non-perfused capillaries | (98, 800) | 469.0449 |
| capVEGFUptakeMax | Max uptake of VEGF-A by capillaries | (8, 32) | 23.8174 |
| MMPsecretionValCap | MMP-9 secreted as capillary is regenerating/sprouting | (4, 16) | 8.2444 |
| NumFibers | Number of fibers nearby must be higher than this to check for capillary distances | (1, 2) | 1.3899 |
| sproutProb | Probability of capillary sprouting behavior | (0.08, 0.32) | 0.1566 |
| secretionUptakeValFibro | Cytokine secretion from fibroblasts per mcs also max uptake value | (0.3, 77) | 9.0436 |
| collagenSecretionValFibro | Collagen secretion from fibroblasts per timestep when it is above ECM | (0.09, 0.24) | 0.1388 |
| distNearSSCdiv | If fibroblast is within this distance of a dividing SSC, it will divide | (5, 65) | 35 |
| TGFActivationThreshold | Level of TGF-β required to activate fibroblast | (20, 80) | 40.3643 |
| TGFMyoThreshold | Level of sustained TGF-β required to turn fibroblast into myofibroblast | (280, 650) | 536.7525 |
| fibroblastTNFApoptosisThreshold | Level of TNF-α to initiate fibroblast apoptosis | (100, 550) | 310.5323 |
| TGFBlockApoptosisThreshold | Level of TGF-β required to block TNF-initiated apoptosis | (160, 1000) | 848.3706 |
| fibroblastApoptosisProb | probability of fibroblast apoptosis if TNF-α and TGF-β conditions are met | (0.65,.99) | 0.8423 |
| IL10decay | Decay rate of IL-10 | (0.00046, 0.00184) | 0.0012 |
| HGFdecay | Decay rate of HGF | (0.000135, 0.0003) | 0.000228 |
| TGFdecay | Decay rate of TGF | (0.0005, 0.05) | 0.0242 |
| TNFdecay | Decay rate of TNF | (0.0000682, 0.002260) | 0.00076272 |
| MCPdecay | Decay rate of MCP-1 | (0.00596, 0.02384) | 0.0124 |
| MMPdecay | Decay rate of MMP-9 | (0.0012, 0.1) | 0.0112 |
| VEGFdecay | Decay rate of VEGF-A | (0.000464, 0.005569) | 0.0015 |
| MinCapDist | Minimum distance between capillary and another capillary | (2.5, 40) | 9.6060 |
| capPlacementThreshold | Threshold for if a capillary will be placed/distance required between fiber and cap | (8, 45) | 34.6346 |
| M2prolifProb | Probability of M2 proliferation behavior occurring | (0.145305, 0.60) | 0.2 |
| M1HalfLife | Corresponds with rate of M1 apoptosis | (15, 55) | 24.6360 |
| M2HalfLife | Corresponds with rate of M2 apoptosis | (30, 70) | 58.1243 |
| reperfusionLag | Probability of capillary regaining perfusion | (0.001, 0.98) | 0.1 |
