# Supplementary material for: Agent-based model demonstrates the impact of nonlinear, complex interactions between cytokines on muscle regeneration
Source: eLife. 2024 Jun 3;13:RP91924. doi: 10.7554/eLife.91924 (PMC11147512; doi:10.7554/eLife.91924)
Supplement: Supplementary file 2. [file elife-91924-supp2.docx]

**Supplemental Table 2.** Cytokine perturbations based on PRCC

| **Perturbation** | **Original cytokine parameter** | **Altered cytokine parameter** |
| --- | --- | --- |
| Decrease HGF decay | 0.000228 | 0.0000776 |
| Increase TGF-β decay | 0.0242 | 0.241899 |
| Increase MMP-9 decay | 0.0112 | 0.101994 |
| Decrease VEGF-A decay | 0.0015 | 0.000249267 |
| Increase MCP-1 diffusion | 0.18627 | 1.76943 |
| Increase MCP-1 decay | 0.0124 | 0.118216 |
| Combination | Above alterations excluding increased MCP-1 decay | |
