# Supplementary material for: Agent-based model demonstrates the impact of nonlinear, complex interactions between cytokines on muscle regeneration
Source: eLife. 2024 Jun 3;13:RP91924. doi: 10.7554/eLife.91924 (PMC11147512; doi:10.7554/eLife.91924)
Supplement: Supplementary file 3. [file elife-91924-supp3.docx]

**Supplemental Table 3.** CPM Mathematical Implementation

| **Representation** | **Mathematical Formulation** | **Equation Terms** | **Explanation** |
| --- | --- | --- | --- |
| Contact Energy (H_1_) | $\sum J\left( \tau_{\left( \sigma\left( i \right) \right),}\tau_{\left( \sigma\left( j \right) \right)} \right)\left( 1-\delta_{\left( \sigma\left( i \right) \right),\left( \sigma\left( j \right) \right)} \right)$ | J | Contact Coefficient |
|  |  | $\sigma\left( i \right)$, $\sigma\left( j \right)$ | Neighboring lattice sites of individual cells |
|  |  | τ | Cell types |
|  |  | $\delta$ | Kronecker delta, localizes contact energy contributions to cell-cell interfaces |
| Volume constraint (H_2_) | $\lambda_{volume}\left( V_{cell} - V_{target} \right)^{2}$ | $\lambda_{volume}$ | Volume constraint scaling factor |
| Logarithmic chemotaxis (H_3_) | $\sum_{c} \frac{\lambda_{c}\left( \tau\left( \sigma\left( y_{i},t \right),t \right) \right)c\left( y_{i},t \right)}{1 + c_{CM}\left( \sigma\left( y_{i},t \right),t \right)}$ | $\lambda_{c}$ | Chemotaxis Parameter |
|  |  | *c* | Chemical field concentration |
|  |  | $c_{CM}$ | Cell body centroid from which chemotaxis behaviors are calculated |
|  |  | τ | Cell types |
|  |  | $t$ | Time |
|  |  | $y_{i}$ | Lattice site |
| Diffusion of chemical species | $\frac{\partial c}{\partial t}=D\nabla^{2}c+kc+S$ | *c* | Chemical field concentration |
|  |  | *k* | Decay constant |
|  |  | *D* | Diffusion constant |
|  |  | *S* | Secretion |
