# Supplementary material for: Agent-based model demonstrates the impact of nonlinear, complex interactions between cytokines on muscle regeneration
Source: eLife. 2024 Jun 3;13:RP91924. doi: 10.7554/eLife.91924 (PMC11147512; doi:10.7554/eLife.91924)
Supplement: Supplementary file 4. [file elife-91924-supp4.docx]

**Supplemental Table 4.** CPM agent adhesion parameters

| **CPM Lattice Interaction** | **Parameter Value** |
| --- | --- |
| Medium - Medium | 0 |
| Medium - Fiber | 0 |
| Medium - ECM | 0 |
| Medium - SSC | 0 |
| Medium - Capillary | 0 |
| Medium - Neutrophil | 0 |
| Medium - Macrophage | 0 |
| Medium - Necrotic | 0 |
| Medium - Wall | 0 |
| Medium - Lymphatic | 0 |
| Fiber - Fiber | 0 |
| Fiber - ECM | 0 |
| Fiber - SSC | 35 |
| Fiber - Capillary | 0 |
| Fiber - Neutrophil | 35 |
| Fiber - Macrophage | 20 |
| Fiber - Necrotic | 0 |
| Fiber - Wall | 0 |
| Fiber - Lymphatic | 0 |
| ECM - ECM | 0 |
| ECM - SSC | 30 |
| ECM - Capillary | 0 |
| ECM - Neutrophil | 30 |
| ECM - Macrophage | 20 |
| ECM - Necrotic | 0 |
| ECM - Wall | 0 |
| ECM - Lymphatic | 0 |
| SSC - SSC | 35 |
| SSC - Capillary | 30 |
| SSC - Neutrophil | 15 |
| SSC - Macrophage | 15 |
| SSC - Necrotic | 30 |
| SSC - Wall | 20 |
| SSC - Lymphatic | 10 |
| Capillary - Capillary | 0 |
| Capillary - Neutrophil | 35 |
| Capillary - Macrophage | 20 |
| Capillary - Necrotic | 0 |
| Capillary - Wall | 0 |
| Capillary - Lymphatic | 0 |
| Neutrophil - Neutrophil | 35 |
| Neutrophil - Macrophage | 15 |
| Neutrophil - Necrotic | 35 |
| Neutrophil - Wall | 20 |
| Neutrophil - Lymphatic | 10 |
| Macrophage - Macrophage | 15 |
| Macrophage - Necrotic | 20 |
| Macrophage - Wall | 20 |
| Macrophage - Lymphatic | 10 |
| Necrotic - Necrotic | 0 |
| Necrotic - Wall | 0 |
| Necrotic - Lymphatic | 0 |
| Wall - Wall | 0 |
| Wall - Lymphatic | 0 |
| Lymphatic - Lymphatic | 0 |
| Fibroblast - Medium | 0 |
| Fibroblast - Fiber | 20 |
| Fibroblast - ECM | 20 |
| Fibroblast - SSC | 15 |
| Fibroblast - Capillary | 20 |
| Fibroblast - Neutrophil | 15 |
| Fibroblast - Macrophage | 15 |
| Fibroblast - Necrotic | 20 |
| Fibroblast - Wall | 20 |
| Fibroblast - Lymphatic | 10 |
| Fibroblast - Fibroblast | 15 |
| Neighbor Order | 4 |
