# Supplementary material for: Agent-based model demonstrates the impact of nonlinear, complex interactions between cytokines on muscle regeneration
Source: eLife. 2024 Jun 3;13:RP91924. doi: 10.7554/eLife.91924 (PMC11147512; doi:10.7554/eLife.91924)
Supplement: Supplementary file 5. [file elife-91924-supp5.docx]

**Supplemental Table 5.** CPM initialization model parameters

| **Parameter** | **Value** | **Justification** |
| --- | --- | --- |
| Simulation lattice size | 321x417x2 | 2 z-layers: Tissue microenvironment layer and migratory cell layer |
| Metropolis Algorithm Temperature | 10 | This is a default, numerically stable temperature for CPM models described in CompuCell3D |
| Neighbor Order | 1 | Interactions spanning 1 pixel distance for Cellular-Potts cell agents was sufficient for agent-based behaviors |
