# Supplementary material for: Agent-based model demonstrates the impact of nonlinear, complex interactions between cytokines on muscle regeneration
Source: eLife. 2024 Jun 3;13:RP91924. doi: 10.7554/eLife.91924 (PMC11147512; doi:10.7554/eLife.91924)
Supplement: Supplementary file 6. [file elife-91924-supp6.docx]

**Supplemental Table 6.** Criteria utilized for CaliPro model calibration

| **Iteration** | **Criteria** |
| --- | --- |
| 1 | End criteria: SSC, M1 macrophages, M2 macrophage, fibroblasts, and neutrophils must be below max threshold at the final timepoint (threshold held constant for all iterations). |
| 2 | SSC, CSA, and end criteria: within 4 times the experimental std for SSC and 1 times CSA std. |
| 3 | Fibroblast, SSC, CSA, replicate check, and end criteria: within 2.1 times the experimental std for fibroblasts, 2.5 for SSC, and 1 times CSA std. More than 1 replicate of the parameter set must meet the criteria. |
| 4 | Fibroblast, SSC, CSA, and end criteria: within 2.2 times the experimental std for fibroblasts and SSC and 1 times CSA std. |
| 5 | Fibroblast, SSC, CSA, and end criteria: within 1.5 times the experimental std for fibroblasts, 1.75 for SSC, and 1 times CSA std. |
| 6 | Fibroblast, SSC, CSA, and end criteria: within 1.75 times the experimental std for fibroblasts, 1.5 for SSC, and 1 times CSA std. |
| 7 | Fibroblast, SSC, CSA, replication, and end criteria: within 2.5 times the experimental std for fibroblasts and SSC, and 1 times CSA std. All replicates are required to pass for the parameter set to pass. |
