# Supplementary material for: Agent-based model demonstrates the impact of nonlinear, complex interactions between cytokines on muscle regeneration
Source: eLife. 2024 Jun 3;13:RP91924. doi: 10.7554/eLife.91924 (PMC11147512; doi:10.7554/eLife.91924)
Supplement: Supplementary file 7. [file elife-91924-supp7.docx]

**Supplemental Table 7.** Experimental data description for model comparison

| **Experimental data description and relevant figure from literature** | **Model comparison** |
| --- | --- |
| CTX injury to mouse hindlimb muscle and quantification of average cross-sectional area (μm^2^) of individual muscle fibers for a given animal was determined after manually outlining individual muscle fibers in each of the digitally captured images (Fig. 3B)^102^ | Muscle CSA recovery calibration (Fig. 3A) |
| BaCl_2_ injury to mouse hindlimb and IF quantification of SSC (Pax7+) and fibroblasts (Tcf4+) cells (Fig 2DD & EE)^76^ | SSC and fibroblast count calibration (Fig. 3B & C) |
| NTX injury to mouse hindlimb and macrophage quantification based on number of F4/80+ cells (Fig. 4E)^97^ | Total macrophage count validation (Fig. 3D) |
| CTX injury to mouse hindlimb muscle and capillary density was calculated using biotinylated lectin and H&E (Fig. 6B)^102^ | Capillaries/mm^2^ validation (Fig. 3H) |
| BaCl_2_ injury to mouse hindlimb and flow cytometry to assess CD45^+^/F4/80^+^/Ly6C^hi^ (M1) and CD45^+^/F4/80^+^/Ly6C^lo^ (M2) macrophages (Fig. 2A)^107^ | M1 and M2 cell count validation (Fig. 3E & H) |
| CTX injury to mouse hindlimb muscle and IHC analysis was used to label neutrophils using Ly6G antibody (Fig. 3A)^108^ | Neutrophil cell count validation (Fig. 3G) |
| Bothrops asper snake venom induced hindlimb injury to mice pretreated with either an antimouse granulocyte rat monoclonal immunoglobulin G (IgG) antibody or with isotype-matched control antibody. Inflammatory infiltrate was quantified from muscle tissue (Fig. 7A) and the extent of regeneration was quantitatively assessed by determining the percentage of the area comprised of regenerating muscle fibers in relation to the total area of muscle damage^109^ | Neutrophil depletion perturbation (Fig. 4) |
| IL-4–conjugated gold nanoparticles were injected into murine skeletal muscle following hindlimb ischemia. M1 and M2 cells were quantified with flow cytometry (Fig. 6 A, C, and E) and muscle force was measured for quantification of regeneration (Fig. 4G)^164^ | Anti-inflammatory nanoparticles perturbation (Fig. 4) |
| IL-10 null mice underwent hindlimb unloading and reloading to induce muscle damage. Muscle CSA (Fig. 6C), M1 and M2 cells (Fig. 3A-B), and SSC differentiation markers were quantified (Fig. 3C-F)^163^ | IL-10 KO perturbation (Fig. 4) |
| Noetoxin or freeze injury to CXCL12Gagtm/Gagtm and control mouse hindlimb muscle with H&E for assessment of muscle regeneration (Fig. 3), transcriptome analysis to asses collagen organization and synthesis (Fig. 2C), and immune cell infiltration (Fig. 4S)^111^ | Hindered angiogenesis perturbation (Fig. 4) |
| Following CTX injury to mouse hindlimb muscle and injection of intramuscular administration of rAAV vectors injury area was quantified for various doses of AAV-VEGF (Fig. 3D) and H&E was used to assess muscle recovery (Fig. 5)^73^ | VEGF-A injection perturbation (Fig. 4) |
| CCL2-/- and control mice received BaCl_2_ injection to induce acute injury. CSA quantification was used to assess muscle regeneration (Fig. 1I). Flow cytometry was used to quantify macrophage (Fig. 2A), and neutrophil (Fig. 2B) cells^115^ | MCP-1 KO perturbation (Fig. 4) |
| CTX induced injury to control and TNF-α receptor double-knockout mice. Force was measured to quantify muscle recovery (Fig. 7), immunohistochemical staining of Mac-1 for quantifying immune cells (Fig. 6B), and western blot analysis was used to assess SSC differentiation (Fig. 2)^165^ | TNF-α KO perturbation (Fig. 4) |
| Mice were treated with clodronate-containing or control liposomes and underwent a contusion injury. H&E was used for muscle regeneration assessment (Fig. 2), HGF (Fig. 4A) and TGF (Fig. 5A) mRNA were quantified^110^ | Macrophage depletion perturbation (Fig. 4) |
